# In-silico and in-vitro screening of Asiatic acid and Asiaticoside A against Cathepsin S enzyme

**DOI:** 10.1186/s40360-023-00701-x

**Published:** 2023-11-25

**Authors:** Temitope Akinwumi Ajani, Kenechukwu Obikeze, Zandisiwe E. Magwebu, Samuel Egieyeh, Chesa G. Chauke

**Affiliations:** 1 University of the Western Cape, School of Pharmacy, Bellville, South Africa; 2 South African Medical Research Council, Primate Unit and Delft Animal Centre (PUDAC), Cape town, South Africa

**Keywords:** Asiatic acid, Asiaticoside a, CatS, Atherosclerosis, *Centella asiatica*

## Abstract

**Background:**

Atherosclerosis is a form of cardiovascular disease that affects the endothelium of the blood vessel. Series of events are involved in the pathophysiology of this disease which includes the breaking down of the connective tissue elastin and collagen responsible for the tensile strength of the arterial wall by proteolytic enzyme. One of these enzymes called Cathepsin S (CatS) is upregulated in the progression of the disease and its inhibition has been proposed to be a promising pharmacological target to improve the prognosis of the disease condition. Asiatic acid and asiaticoside A are both pentacyclic triterpenoids isolated from *Centella asiatica*. Their use in treating various cardiovascular diseases has been reported.

**Methods:**

In this study through in silico and in vitro methods, the pharmacokinetic properties, residue interaction, and inhibitory activities of these compounds were checked against the CatS enzyme. The SwissADME online package and the ToxTree 3.01 version of the offline software were used to determine the physicochemical properties of the compounds.

**Result:**

Asiatic acid reported no violation of the Lipinski rule while asiaticoside A violated the rule with regards to its molecular structure and size. The molecular docking was done using Molecular Operating Environment (MOE) and the S-score of − 7.25988, − 7.08466, and − 4.147913 Kcal/mol were recorded for LY300328, asiaticoside A, and asiatic acid respectively. Asiaticoside A has a docking score value (− 7.08466Kcal/mol) close to the co-crystallize compound. Apart from the close docking score, the amino acid residue glycine69 and asparagine163 both interact with the co-crystallized compound and asiaticoside A. The in vitro result clearly shows the inhibitory effect of asiaticoside and asiatic acid. Asiaticoside A has an inhibitory value of about 40% and asiatic acid has an inhibitory value of about 20%.

**Conclusion:**

This clearly shows that asiaticoside will be a better drug candidate than asiatic acid in inhibiting the CatS enzyme for the purpose of improving the outcome of atherosclerosis. However, certain modifications need to be made to the structural make-up of asiaticoside A to improve its pharmacokinetics properties.

**Supplementary Information:**

The online version contains supplementary material available at 10.1186/s40360-023-00701-x.

## Background

Atherosclerosis is a form of cardiovascular disease characterized by inflammation and occurs in the blood vessel due to the distortion of the endothelium lining of the artery by fatty streak deposition [1]. It results in the development of plaque and arterial vessel wall remodeling. This disease is responsible for one-third of all death due to cardiovascular disease worldwide [2]. (In atherosclerosis disease, the protein components of the extracellular matrices (collagen, elastin, and aminoglycan) responsible for the rigidity, tensile strength, and stability of the artery are degraded by proteolytic enzymes including Cathepsin S (CatS) [3, 4]. Studies have reported an upregulation of the expression of CatS in subjects with atherosclerosis when compared to non-atherosclerosis subjects [5–8]. In the homeostasis state, CatS enzyme activity is regulated by the endogenous enzyme cystatin C, which is produced at the same rate as CatS to regulate the extent of degradation of elastin, collagen, and aminoglycan in the extracellular matrices [9, 10]. However, in atherosclerosis, the upregulation of CatS is not followed by the upregulation of cystatin C, thereby leaving an excess of CatS which participate in the degradation of the extracellular matrixes. This suggests that the exogenous inhibition of CatS could be a promising venture in the management of atherosclerosis and a few studies have suggested this claim [11–13]. Since the crystal structure was elucidated [14] and the establishment of its involvement in the pathophysiology of diseases like cancer [15, 16], cardiovascular disease [17], autoimmune disease [18] there has been effort to identify an inhibitor for CatS with various challenges associated with the complexity of the crystal structure and the number of cathepsin isomers available [19–21].

Plants have been a source of drugs for a long time and the process of developing promising drugs from plants based on ethnobotanical and ethnopharmacological claims is a cumbersome and costly one. However, with the advent of the fourth industrial revolution and the use of molecular techniques like the in silico method this process has been made simple [22]. The medicinal plant *Centella asiatica* (Linn.) Urban is used in various traditional formularies for the treatment of a wide variety of diseases and has been reported to be used for the management of cardiovascular disease [23, 24]. Pentacyclic triterpenes have been reported as the main secondary metabolites responsible for the pharmacological effects of *C. asiatica.* Pentacyclic triterpenoids are a class of chemical compounds occurring as free acids, esters, or glycosides from natural plant materials [25]. Pentacyclic triterpenoids have been studied for their anti-inflammatory actions, hypoglycemic action, antibacterial actions, antioxidant action, antitumor activities, antiproliferative activities, anti-HIV, cardiovascular, and a lot more [23, 26]. The pentacyclic triterpenoids asiatic acid and asiaticoside A (madecassoside) have been reported as exhibiting anti-inflammatory, anticancer, cardioprotective, and neuroprotective effects [23, 27–29]. This study employed molecular docking to predict the interactions between CatS and the pentacyclic triterpenoids asiatic acid and asiaticoside A. Fluorescent-based enzyme inhibition assay was utilized to evaluate the predicted interactions between the enzyme and compounds in vitro.

## Methods

This study was a basic research in the identification of a CatS enzyme inhibitor. The test compounds (asiatic acid and asiaticoside A) were purchased from Sigma-Aldrich, South Africa, while the cathepsin S inhibitor Assay kit (Fluorometric-ab185437) was purchased from Abcam (South Africa). The Cathepsin S inhibitor assay kit contained cathepsin S enzyme, cathepsin S inhibitor (Z-FF-FMK, 1 mM), cathepsin S substrate (Z-VVR-AFC), and a physiological buffer solution. Greiner 96 Black Flat Bottom Fluotrac plates were used for the experiment as recommended by the kit manufacturer.

## Physicochemical and pharmacokinetic characterization of asiatic acid, and asiaticoside a

The canonical Simplified molecular-input line-entry system (SMILES) sequence of asiatic acid and asiaticoside A were copied from PubChem [30] and pasted to SwissADME to determine their physicochemical and pharmacokinetics properties according to the method described by Daina, Michielin [31] Toxtrees a GUI application that can estimate the toxic hazard of chemicals was used to analyze the carcinogenicity and mutagenicity of the compounds [32, 33].

## Ligand preparation

The molecular interaction between the ligands and the receptor complex was studied with the molecular docking software Molecular Operating Environment (MOE 2016.01). The structures of asiatic acid and asiaticoside A (madecassoside) were searched and downloaded from the PubChem online database in standard data format (.sdf) and converted to the “. mol” format with OpenBabel (version 3.1.1) [34]. The “. mol” format of the ligands was imported into the MOE software and was protonated, and energy minimized with MMFF94x force field at an energy gradient of 0.001 kcal/mol. A ligand database was then created, and the two compounds (asiatic acid and asiaticoside A) were saved in the ligand database in “.mdb” format.

## Molecular docking

The three-dimensional crystal structure of CatS with its co-crystallized compound (LY3000328) was downloaded from the Research Collaboratory for Structural Bioinformatics (RCSB) website (PDB: 4P6G) [11]. MOE was used to determine the molecular interaction between the ligand and the three-dimensional structure of the enzyme- CatS. MOE protein module was used in the preparation of the enzyme for virtual screening. The binding site was defined as the area occupied by the co-crystalline compound (ligand). The enzyme was protonated, washed and energy was minimized at an energy gradient of 0.001 kcal mol-1, and docking was carried out and the results were analysed for binding affinity, molecular interactions as described previously [35].

## In-vitro cathepsin S inhibition assay

Cathepsin enzyme was diluted with 100 μl of the physiological buffer solution and aliquoted into 20 μl portion in a 500 μl Eppendorf tube and stored in the − 80 °C fridge until it was used as recommended by the manufacturer. To evaluate the inhibition of cathepsin S activity by asiatic acid and asiaticoside A, 5 concentrations (0.1 μg/ml, 1 μg/ml, 10 μg/ml, 20 μg/ml, and 50 μg/ml) of the compounds were prepared from a 1 mg/ml stock solution, 10 μl of each concentration was pipetted into to a 96 well plate in triplicate. An enzyme mix (1 μl of CatS and 49 μl of buffer) was added to each well before incubating the mixture for 15 minutes, protected from light and at room temperature. Following incubation, the substrate mix (2 μl of substrate and 38 μl of buffer) was added to the plate. Three (3) control reactions were prepared. A blank (containing the buffer alone), an enzyme-substrate reaction (containing the CatS enzyme, Z-VVR-AFC, and physiological buffer solution), and the inhibitor reaction (containing CatS, Z-FF-FMK, and the physiological buffer solution). The 96-well plate was then transferred to the fluorescent plate reader for fluorescence reading. Excitation and emission wavelengths were set at 400 and 505 respectively and readings were done every minute for 60 minutes in kinetic mode at 37 °C. The percentage of relative inhibition was calculated from the slopes of the graphs using the formula below

$$\mathit\%\,\textit{Relative}\mathit\;\textit{Inhibition}\mathit=\frac{\textit{Slope}\mathit\;\textit{of}\mathit\;\textit{enzyme}\mathit\;\textit{control}\mathit-\textit{Slope}\mathit\;\textit{of}\mathit\;\textit{Sample}}{\textit{Slope}\mathit\;\textit{of}\mathit\;\textit{enzyme}\mathit\;\textit{control}}\mathit\times\mathit{100}$$

## Statistical analysis

Experiments were done in triplicate; results were analyzed and percentage inhibition was determined with Microsoft Excel 365 and the graphs of average fluorescence with time were plotted for each concentration using GraphPad prism 8.0.

## Result

Physicochemical and pharmacokinetics properties of asiatic acid and asiaticoside A were determined by the SwissADME predictor and Toxtree software shown in Table 1. The basic information represents the parameters for the Lipinski rule of five in estimating a drug candidate. The molecular weight, number of hydrogen bond acceptor/donor, and the number of rotatable bonds were presented in Table 1. The octanol-water partition coefficient (cLogP) was calculated as an average of 5 values from different models.

**Table 1:**
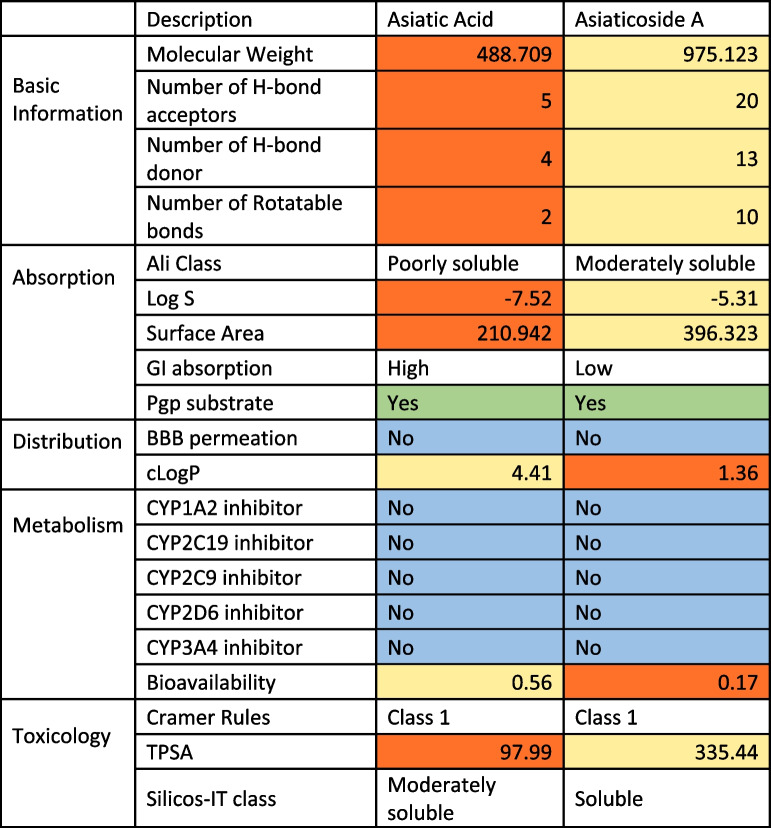
Physicochemical and pharmacokinetics properties of asiatic acid and asiaticoside A

(GI-Gastrointestinal; Pgp- Permeability Glycoprotein; BBB- blood brain barrier; cLogP- partition coefficient; Log S- aqueous solubility; CYP- cytochrome P450 enzyme; TPSA- Topological Polar Surface Area). According to Lipinski rule and other variant, a potential drug for oral administration should have at least 5 Hydrogen bond donors, 10 Hydrogen bond acceptors, molecular weight less than 500, cLogP less than 5, at least 10 rotatable bonds, less than 140Å^2^ surface area [31]

The pharmacokinetic properties of the compounds were represented as absorption, distribution, metabolism, excretion, and toxicology (ADMET). The absorption of the compounds was determined by Ali class which measure the solubility of the compounds, Gastrointestinal absorption which was a measure of the absorption of the compound in the gastrointestinal track and the permeability glycoprotein (Pgp) which measure the permeability of the compound across the cells and membranes.

The drug distribution was determined by the blood brain barrier distribution and cLogP which was the logarithm of the partition coefficient of the n-octanol and water which was also a measure of the drug hydrophobicity. cLog P is also a factor in the Lipinski law of 5. Drug metabolism was determined by how the drug interact with the cytochrome p450 enzyme. The cramer rules was used to determine the drug toxicity.

## Molecular docking

When docked with the CatS enzyme, LY3000328 and asiaticoside A had similar docking scores (− 7.25988 and − 7.08466 Kcal/mol, respectively), while asiatic acid reported a much larger docking score (− 4.147913 Kcal/mol). The native ligand LY3000328 interacted with four amino acid residues (Gly69, Asp163, Lys64, and Glu115) and Asiaticoside A also interacted with four amino acid residues (Gly69, Cys25, Asp163, and Gly23). LY3000328 and asiaticoside A had two common amino acid (Gly69 and Asp163) that interact with CatS enzyme. Asiatic acid on the other hand interacted with only one amino acid residue (Met 71) which was not similar to the amino acid interactions for LY3000328 and asiaticoside A (Table 2).

**Table 2:** Molecular docking scores and amino acid residue interactions for LY3000328, asiaticoside A and asiatic acid

S/N	Ligand	Docking score (Kcal/mol)	Amino acid residue interaction/Type interaction
1	CatS Inhibitor LY3000328	−7.25988	N28 - GLY 69 /H-donor N29 -ASN 163 / H-donor O31- GLY 69/ H-acceptor F35-LYS 64/ H-acceptor N27-GLU115/ ionic
2	Asiaticoside A (Madecassoside)	−7.08466	O13-GLY 69/ H-donor O16-CYS 25/ H-donor C61-ASN 163/ H-donor O18-GLY 23/ H-acceptor
3	Asiatic Acid	−4.147913	O1-MET 71/ H-donor O3-MET 71/ H-donor

The co-crystallized compound (LY3000328) and asiaticoside A interact with amino acid residues Gly69 and Asp163 of the CatS enzyme. Asiatic acid, on the other hand, does not interact with any amino acid residue in-common with the co-crystallized compound.

**Fig. 1:**
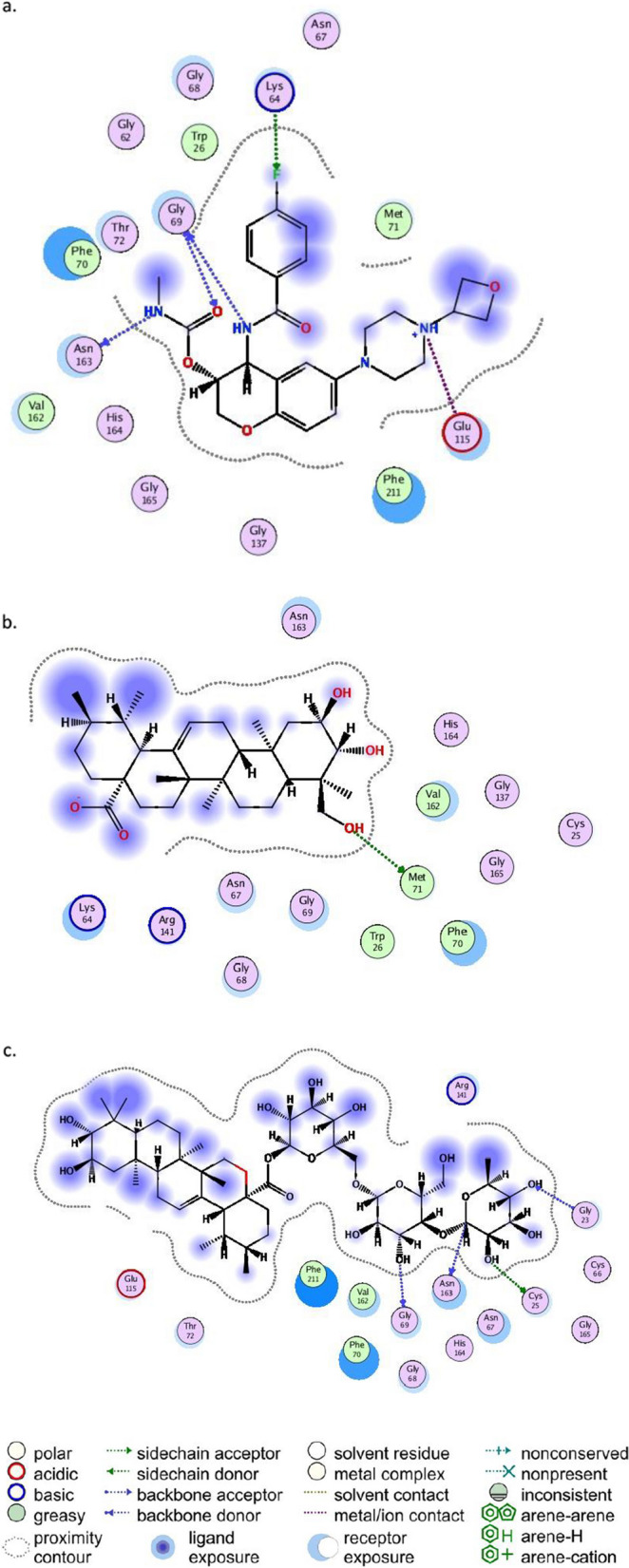
2D Interactions between (**a**) LY3000328, (**b**) asiatic acid and (**c**) asiaticoside A and amino acid residues of the CatS active site. The green region represent H-bond acceptor and pink region represents H-bond donor. H-bond interactions are shown with green bond and hydrophobic interactions are represented with other colors

The interaction between the amino acid residue of CatS with LY3000328, Asiatic acid, and asiaticoside are represented in Fig. 1a, b, and c, respectively. LY3000328 had three hydrogen bond interactions with Gly69, Asn163, and Lys64 and an ionic bond interaction with GLU115. Asiatic acid interacted with Met 71 via a hydrogen bond while asiaticoside A interacted with Phe211, Glu115, Asn67, and Arg141 via hydrogen bonds and with Phe211 via a pi bond (Fig. 1).

## In-vitro cathepsin S inhibition assay

A percentage inhibition for each compound was calculated with the formula discussed earlier. The inhibition percentage was plotted on the y axis and different concentrations (0.1 μg/ml, 1 μg/ml, 10 μg/ml, 20 μg/ml, and 50 μg/ml) were plotted on the x-axis. Asiaticoside A and asiatic acid inhibit CatS enzyme in a dose dependent pattern.

## Discussion

Evaluation of the pharmacokinetic properties of a drug experimentally is very expensive and time-consuming so the use of the computational method is better in saving cost and time [22]. Drug lipophilicity, pharmacokinetics, water-solubility, drug-likeness, and medicinal chemistry properties are considered and reported in Table 2. The pharmacokinetic properties of a drug compound are the most important in computational drug development because it is the major factor to consider before a compound can become a drug candidate. While the binding force and the scoring function of a compound are important, it is also important that the compound can reach the target organ at therapeutically effective concentration/ dose, and all of these are determined by the pharmacokinetic properties of the drug compound. The pharmacokinetic properties of a drug include the drug absorption, distribution in the body, metabolism of the drug, excretion, and toxicology of the drug. The molecular weight of the compound is important because the mode of the entrance of most drugs into the cell and tissues is by simple diffusion and a drug with a very high molecular size might have to depend on other macromolecules to enter the cells and tissues. Therefore, a compound with a very high molecular weight might not be ideal for a drug candidate.

Ali classification, Log S, surface area, GI absorption, Pgp substrate (e.g digoxin, fexofenadine, ambrisentan etc. and inhibitors (e.g itraconazole dronederone larithromycin etc) are all factors that affect drug absorption. Going by the Ali classification Asiatic acid is slightly soluble while asiaticoside A is moderately soluble. Log S talks about the water solubility of the compounds. The water solubility of the compounds is determined by different models (log S, Ali Solubility, ESOL solubility, and Silicos-IT Solubility) with no specific consensus. All the results for the water solubility of the compounds were reported and asiatic acid looks more soluble in water than asiaticoside A. Permeability glycoprotein (Pgp) is a drug transporter that plays a very critical role in the pharmacokinetics of drugs [36]. Its ability to act as a drug transporter should be put into consideration when a new drug is being developed. Both Asiatic acid and asiaticoside A are substrates for Pgp and this implies that their absorption and bioavailability can be affected by this glycoprotein., Asiatic acid and asiaticoside A should be given with caution especially when other drugs are given because of drug-drug interaction which can lead to the induction or inhibition of the Pgp. Pgp is a substrate of Asiatic acid and asiaticoside A which implies that this substrate plays a part in the transportation of these compounds across the membrane. Therefore, any drug that induces this substrate will increase the rate of transportation of the compounds and reduce the bioavailability of the compound as it is known to act as an efflux pump that pumps the drug back into the lumen.

Applying Lipinski’s rule of 5 to the drug molecule can be beneficial and give a great insight into drug distribution. The cLogP of a compound aimed to be used as oral drugs is expected to be less than 5. The cLogP is a measure of the relative solubility of the compound in water and biological environment. It is determined by measuring the partition coefficient of the compound between octan-1-ol and water. This value is expected to be between 0.5 and 5 [37]. The cLogP of Asiatic acid (4.41) and asiaticoside A (1.36) are less than 5 so according to cLogP predictions, these compounds can be considered a drug candidate for oral administration.

The drug-likeness of the compounds was assessed by the Abbot Bioavailability Score and five different rule-based filters embedded in the SwissADME application which include Lipinski from Pfizer, Ghose from Amgen, Veber from GSK, Egan from Pharmacia, and Muegge from Bayer. The Lipinski rule was reported here to establish the drug-likeness of the compounds. According to Daina, Michielin [31], the bioavailability score is dependent on the total charge on the compound, TPSA, and violation of the Lipinski rule. This classified drugs into 4 bioavailability groups which are 11, 17, 56, and 85% with drugs in the 85% group being the most bioavailable and the drug in the 11% group being the least bioavailable. The SwissADME also measures the bioavailability of drug administered through oral means and 56 and 17% is determined for Asiatic acid and Asiaticoside A respectively. Which suggests that Asiatic acid will be more available in the plasma compared to asiaticoside A when they are both administered orally because of the first pass effect.

The metabolism of the compound was also predicted by the SwissADME tool. The two drugs are not metabolized by CYP1A2, CYP2C1, CYP2C9, CYP2D6, and CYP3A4, which are responsible for the polymorphism in the metabolism of drugs. Toxtree software predicts the mutagenicity and carcinogenicity of a ligand/compound by examining the molecular structure of the compounds. The Toxtree 3.01 categorizes drug compounds into 3 categories, and it has been reported that its prediction is 75.8% accurate [38]. It is based on the Cramer rules which ask a different question about the chemical structure of a ligand before classifying a ligand as a class I,Class II or Class III compound. Class I compounds have low mutagenicity and carcinogenicity values. Class II compounds are more complex compared to class I compounds. And they are classified as intermediate as far as mutagenicity and carcinogenicity are concerned. Class III compounds are classified to have high possibility of carcinogenicity and mutagenicity. This classification is done based on the presence of reactive or presence of toxic functional groups. Both Asiatic acid and asiaticoside A are class I compounds which suggest that they are not mutagenic or carcinogenic, so they are safe to be considered as a drug candidate.

The docking score is the measure of the binding affinity calculated in Kcal/mol, the lower the binding affinity, the better the docking score. So, ligand-receptor docking that has lower binding affinity are preferred and have more prospects in the process of drug development. The docking score of LY3000328 is − 7.3Kcal/mol, asiaticoside A − 7.1Kcal/mol, and Asiatic acid a docking score of − 4.1Kcal/mol. The docking score of asiaticoside A is very close to the score of the co-crystalized compound and the residue interaction suggests that it also has good interaction with the amino acid residue of the CatS enzyme. GLY69 and ASN163 are both amino acid residues that LY3000328 and asiaticoside A interact to form a hydrogen bond. Asiaticoside also interacts with Cys25 which is one of the amino acids that form the triad of the active site of the CatS enzyme [14]. There is a hydrogen bond between O16 on asiaticoside A and CYS25 on CatS enzyme to form a covalent bond. All these suggest that asiaticoside A can be a more active inhibitor of CatS in atherosclerosis situations.

Ligand interaction, docking score, and the physicochemical properties of a ligand are all important and must be observed and considered holistically. An ideal ligand for a drug candidate must pass on all fronts.. From the in silico result, asiaticoside A has a better scoring function than Asiatic acid despite the pharmacokinetics advantages of Asiatic acid as stated above. The amino acid residue at the S2 (Phe70, Gly137, Val162, Gly165, and Phe211) and S3 (Gly62, Asn63, Lys64, Gly68, Gly69, and Phe70) pocket of the cathepsin enzyme is said to be responsible for the enzyme specificity and compounds interacting with amino acid at this center are likely to be specific for cathepsin S as against other cathepsins isoforms [14]. Lack of specificity has been a major setback in the development of CatS inhibitor [39]. From Fig. 1a and b, LY3000328 and asiaticoside A interacted with amino acid residue at the S3 pocket. The amino acid residue on the S3 pocket that LY3000328 interact with are Gly69, and Lys64, and the amino acid residue at the S3 pocket that asiaticoside A interact with is Gly69. However, the interaction with Cys25 is a very important interaction that suggest and confer potency on a compound as a covalently bond inhibitor. This suggest that asiaticoside is a covalent inhibitor of cathepsin S [40].

**Fig. 2:**
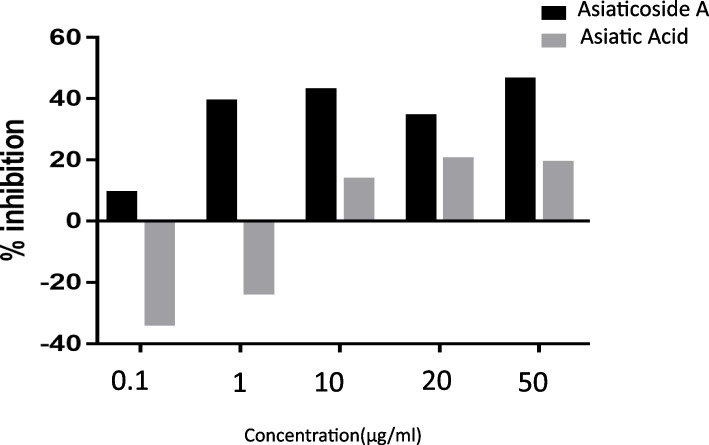
Relative percentage inhibition of cathepsin S enzyme activity by asiatic acid and asiaticoside A **(**0.1 μg/ml, 1 μg/ml, 10 μg/ml, 20 μg/ml, and 50 μg/ml)

Both compounds (asiatic acid and asiaticoside A) were tested in vitro, Fig. 2 shows that they were both inhibitory to the enzyme in a dose-dependent manner. A very low concentration of asiaticoside A (0.1 μg/ml) restricted the enzyme and a 10-fold increase in the concentration of asiaticoside A to 1 μg/ml caused a very significant change in enzyme inhibition as seen on the chart. A further 10-fold increase in concentration does not show the same pattern of inhibition observed earlier and this indicates that at low concentrations asiaticoside A is more active as an inhibitor of CatS than at high concentration. This might just be because of the molecular size of the compound. Increasing the concentration further increases its inhibitory activities but is not as significant as compared to what was observed at lower concentration. Asiatic acid, on the other hand, has no inhibitory effect on CatS at low concentrations (Fig. 2). In fact, increasing the initial concentration by 10fold does not correspond to an increase in inhibitory activities. However, at high concentrations 10 μg/ml, it shows an inhibitory effect against the CatS enzyme and further increase in the concentration of asiatic acid also increases the inhibitory activities of CatS. Furthermore, asiaticoside A reaches a climax in its inhibition of CatS at 40% and an increase in concentration beyond that point does not result in increase in inhibition. It then shows that Asiaticoside A is a better inhibitor of CatS at lower concentration than when the concentration is increased. This also shows a dose-independent inhibition of CatS as it reaches a maximum inhibition of 20%. Asiatic acid shows little or no inhibition of CatS at lower concentration. An increase in concentration of asiatic acid results in an increase in inhibition but the highest inhibition value of this enzyme is 20% even at high concentration. The pharmacokinetic properties of a drug, its molecular interaction, binding scores, and biological actions are all very important. Therefore, an ideal drug compound would be one that can check all the boxes of this criteria. Both compounds seem not to be suitable as a drug candidate but asiaticoside A looks more promising if the molecular structure is modified.

The IC_50_ which determines a drug potency is a measure of the concentration of a drug compound that can cause 50% inhibition of the CatS enzyme activities. The IC_50_ of asiatic acid and asiaticoside A were determined as 14.63 and 0.667 μM respectively. The IC_50_ value suggest that asiaticoside A because of its lower IC_50_ compare with asiatic acid might be a better drugs candidate in inhibiting CatS enzyme but its potency cannot be compare with LY3000328 with an IC_50_ of 7.70 nM. Hence there is a need to improve the potency of asiaticoside A in order to consider it as a drug candidate.

## Conclusion

This study has demonstrated the interaction of two inhibitors with CatS enzymes. Asiaticoside A shows a better molecular interaction, docking score,inhibitory properties and IC_50_ compared with asiatic acid. This indicates that it might be a better compound to inhibit the activities of CatS in the progression of atherosclerosis and other related diseases. However, the physiochemical properties of asiaticoside A do not favor it to be considered as a drug candidate because of violating certain principles, its docking scores and pharmacological inhibition of CatS make it more suitable to be considered as a right candidate for inhibition of CatS enzyme. This suggests that the compound needs to be modified to meet all the criteria suitable for a drug candidate and the test can be repeated to ascertain the suitability of the compound.

### Supplementary Information

**Additional file 1.**

## Data Availability

We have shared the raw data by providing it in a supplementary file.

## Abbreviations

ADMET: Absorption, Distribution, Metabolism, Excretion, Toxicity
Asn: Asparagine
BBB: Blood Brain Barrier
CatS: Cathepsin S
cLogP: Partition Coefficient
CYP: Cytochrome P450
Cys: Cysteine
GI: Gastrointestinal
Gly: Glycine
Log S: Aqueous Solubility
Lys: Lysine
Pgp: Permeability glycoprotein
Phe: Phenylalanine
TPSA: Topological Polar Surface Area
Val: Glycine
