# Supplementary material for: In-silico and in-vitro screening of Asiatic acid and Asiaticoside A against Cathepsin S enzyme
Source: BMC Pharmacol Toxicol. 2023 Nov 25;24:67. doi: 10.1186/s40360-023-00701-x (PMC10676574; doi:10.1186/s40360-023-00701-x)
Supplement: Supplementary file 1 — Additional file 1. [file 40360_2023_701_MOESM1_ESM.docx]

**Table S1. Asiaticoside A % inhibition calculation**

| Asiaticoside A inhibition | | | |
| --- | --- | --- | --- |
| conc µg/ml | enzyme slope | slope (M) | Asiaticoside A % inhibition |
| 0.1 | 827.8 | 752.1 | 9.144721 |
| 1 | 827.8 | 505.31 | 38.95748 |
| 10 | 827.8 | 474.48 | 42.68181 |
| 20 | 827.8 | 545.07 | 34.15439 |
| 50 | 827.8 | 445.89 | 46.13554 |
| 1000 | 827.8 | 1090.7 | -31.7589 |

**Table S2. Asiatic Acid % inhibition calculation**

| Asiatic Acid inhibition | | | |
| --- | --- | --- | --- |
| Conc µg/ml | enzyme slope | Slope (M) | Asiatic Acid % inhibition |
| 0.1 | 827.8 | 1105.1 | -33.4984 |
| 1 | 827.8 | 1019.8 | -23.194 |
| 10 | 827.8 | 716.23 | 13.47789 |
| 20 | 827.8 | 660.69 | 20.18724 |
| 50 | 827.8 | 670.1 | 19.0505 |
| 2000 | 827.8 | 476.36 | 42.4547 |

**Table S3. Asiatic Acid % inhibition and Asiaticoside A % inhibition**

| Conc µg/ml | Asiaticoside A % inhibition | Asiatic Acid % inhibition |
| --- | --- | --- |
| 0.1 | 9.144721 | -33.4984 |
| 1 | 38.95748 | -23.194 |
| 10 | 42.68181 | 13.47789 |
| 20 | 34.15439 | 20.18724 |
| 50 | 46.13554 | 19.0505 |
| 1000 | -31.7589 | 42.4547 |

**Table S4 SwissADME result**

| **Molecule** | **Asiatic Acid** | **Asiaticoside A** |
| --- | --- | --- |
| Formula | C30H48O5 | C48H78O20 |
| MW | 488.7 | 975.12 |
| #Rotatable bonds | 2 | 10 |
| #H-bond acceptors | 5 | 20 |
| #H-bond donors | 4 | 13 |
| MR | 139.24 | 235.98 |
| TPSA | 97.99 | 335.44 |
| iLOGP | 3.2 | 1.87 |
| XLOGP3 | 5.7 | -1.24 |
| WLOGP | 5.03 | -2.06 |
| MLOGP | 4.14 | -2.84 |
| Silicos-IT Log P | 3.96 | -2.53 |
| Consensus Log P | 4.41 | -1.36 |
| ESOL Log S | -6.33 | -4.44 |
| ESOL Solubility (mg/ml) | 2.29E-04 | 3.50E-02 |
| ESOL Solubility (mol/l) | 4.69E-07 | 3.59E-05 |
| ESOL Class | Poorly soluble | Moderately soluble |
| Ali Log S | -7.52 | -5.31 |
| Ali Solubility (mg/ml) | 1.46E-05 | 4.79E-03 |
| Ali Solubility (mol/l) | 2.99E-08 | 4.91E-06 |
| Ali Class | Poorly soluble | Moderately soluble |
| Silicos-IT LogSw | -4.28 | 1.52 |
| Silicos-IT Solubility (mg/ml) | 2.59E-02 | 3.20E+04 |
| Silicos-IT Solubility (mol/l) | 5.31E-05 | 3.28E+01 |
| Silicos-IT class | Moderately soluble | Soluble |
| GI absorption | High | Low |
| BBB permeant | No | No |
| Pgp substrate | Yes | Yes |
| CYP1A2 inhibitor | No | No |
| CYP2C19 inhibitor | No | No |
| CYP2C9 inhibitor | No | No |
| CYP2D6 inhibitor | No | No |
| CYP3A4 inhibitor | No | No |
| log Kp (cm/s) | -5.23 | -13.13 |
| Lipinski #violations | 0 | 3 |
| Ghose #violations | 3 | 4 |
| Veber #violations | 0 | 1 |
| Egan #violations | 0 | 1 |
| Muegge #violations | 1 | 5 |
| Bioavailability Score | 0.56 | 0.17 |
| PAINS #alerts | 0 | 0 |
| Brenk #alerts | 1 | 1 |
| Leadlikeness #violations | 2 | 2 |
